# Early caudate involvement marks a Lewy body spectrum phenotype in parkinsonism: a 5-year longitudinal study

**DOI:** 10.3389/fneur.2026.1895109

**Published:** 2026-10-16

**Authors:** Sara Varanese, Maria Pia Buongarzone, Stefano Viola, Maria Felice Ghilardi, Alessandro di Rocco, Rino Speranza, Alessia Masotino, Christian Durante, Laura Bonanni

**Affiliations:** 1 Neurology Clinic, San Pio Hospital, Vasto, Italy; 2 CUNY School of Medicine, New York, NY, United States; 3 Department of Neurology, Zucker School of Medicine, Hofstra/Northwell Health, New York, NY, United States; 4 Department of Medicine and Aging Sciences, University G. d'Annunzio of Chieti-Pescara, Chieti, Italy

**Keywords:** caudate denervation, cognitive decline, DAT imaging, Lewy bodies dementia, Parkinson’s disease

## Abstract

**Background:**

Parkinsonian syndromes are increasingly recognized as part of a heterogeneous spectrum of Lewy body diseases, ranging from Parkinson’s disease (PD) to dementia with Lewy bodies (DLB). Identifying early markers of clinical heterogeneity remains challenging.

**Objective:**

To investigate whether striatal dopaminergic denervation patterns on DAT-SPECT are associated with distinct clinical phenotypes and long-term cognitive trajectories in early parkinsonism.

**Methods:**

Twenty-eight patients with early parkinsonism fulfilling clinical diagnostic criteria for PD underwent DAT-SPECT and were classified according to the topography of dopaminergic denervation into isolated putaminal involvement (P group) or combined putaminal and caudate involvement (PC group). Motor, neuropsychiatric, and cognitive assessments, including the MoCA, were performed at baseline and after 5 years.

**Results:**

At baseline, patients with caudate involvement (*n* = 16) showed greater motor impairment and a higher prevalence of psychiatric symptoms than the P group. Early cognitive impairment was observed in 32% of the PC group versus 0% of the P group. Over 5 years, the PC group showed greater cognitive deterioration, with 83% scoring below the cognitive impairment threshold compared with 9% in the P group. Visual hallucinations developed in a subset of PC patients during follow-up.

**Conclusion:**

Early caudate dopaminergic denervation identifies a parkinsonian phenotype associated with greater motor severity, neuropsychiatric manifestations, and accelerated cognitive decline. Its longitudinal evolution may reflect a trajectory toward the cognitive and neuropsychiatric end of the Lewy body disease spectrum, including a DLB-like phenotype. DAT-SPECT may provide prognostic information beyond its diagnostic utility.

## Introduction

Parkinsonian syndromes are increasingly recognized as part of a broader spectrum of neurodegenerative disorders characterized by α-synuclein pathology, collectively referred to as Lewy body diseases. Within this continuum, Parkinson’s disease (PD) and dementia with Lewy bodies (DLB) share overlapping clinical, neuropsychological, and neuropathological features, differing primarily in the temporal sequence and relative prominence of motor and cognitive symptoms (1). In clinical practice, however, distinguishing PD from early DLB remains challenging, particularly in patients presenting with cognitive impairment or neuropsychiatric manifestations (1, 2).

Beyond the classical motor symptoms of parkinsonism—bradykinesia, rigidity, tremor, and postural instability—nonmotor features such as cognitive decline, mood disorders, sleep disturbances, and psychosis are increasingly recognized as major determinants of disability, reduced quality of life, and disease progression (2–5). Neuropsychiatric symptoms, including depression, apathy, and psychosis, have been consistently associated with cognitive impairment and poorer prognosis in PD, suggesting a more widespread neurodegenerative process beyond the nigrostriatal system (2, 6, 7).

The topographical distribution of dopaminergic degeneration within the striatum has been proposed as a key factor underlying clinical heterogeneity in parkinsonian disorders. Neuropathological studies have demonstrated an uneven pattern of dopamine depletion within the striatum, with more pronounced involvement of the putamen compared to the caudate nucleus in early PD (6). Functionally, these structures are embedded within distinct cortico-striatal circuits: the putamen is primarily involved in motor control, whereas the caudate nucleus is part of associative and limbic loops subserving executive, cognitive, and behavioral functions (8). In line with this organization, neuroimaging studies have shown that dopaminergic dysfunction in the caudate correlates with impaired executive function and global cognitive performance in PD (8–10). Additional PET and SPECT studies have demonstrated that caudate dopaminergic denervation may occur early in PD and is strongly associated with executive dysfunction, global cognitive impairment, and early cognitive vulnerability (11–13). Moreover, structural and functional alterations in fronto-striatal and limbic networks have been linked to psychiatric manifestations, including depression and apathy (7, 14, 15).

Importantly, emerging evidence suggests that early involvement of the caudate nucleus may identify patients with a more aggressive disease trajectory. Longitudinal imaging studies indicate that dopaminergic dysfunction extending beyond the putamen to the caudate is associated with faster cognitive decline and increased risk of dementia (9, 10). These observations support the hypothesis that the topographical pattern of striatal degeneration may reflect the extent and progression of the underlying synucleinopathy, potentially positioning patients along a continuum between PD and DLB.

Dopamine transporter single-photon emission computed tomography (DAT-SPECT) using [123I]FP-CIT provides an *in vivo* measure of presynaptic dopaminergic integrity within the striatum and is widely used in the diagnostic workup of parkinsonian syndromes (16). While DAT-SPECT has high sensitivity and specificity for detecting nigrostriatal degeneration, it cannot reliably differentiate between PD and other Lewy body diseases such as DLB (1, 16). Nevertheless, it offers a valuable opportunity to characterize patterns of striatal involvement that may have prognostic implications.

Despite growing interest in imaging biomarkers, the longitudinal clinical significance of specific striatal dopaminergic patterns—particularly early caudate involvement—remains insufficiently explored. Most available studies are cross-sectional or focus on global dopaminergic deficits, leaving a gap in understanding how regional patterns of denervation relate to long-term clinical outcomes.

In the present study, we investigated the clinical and cognitive correlates of striatal dopaminergic denervation patterns in a cohort of patients with early parkinsonism. Specifically, we aimed to determine whether patients with combined putaminal and caudate involvement exhibit a distinct clinical phenotype at baseline and a different cognitive trajectory over a 5-year follow-up period. We hypothesize that early caudate dopaminergic denervation would identify a subgroup of patients with a more severe and rapidly progressive clinical profile, characterized by early cognitive impairment and features overlapping with the Lewy body disease spectrum.

## Methods

### Participants

Twenty-eight patients diagnosed with Parkinson’s disease based on the Movement Disorder Society (MDS) clinical diagnostic criteria were consecutively recruited from the Parkinson and Movement Disorders Center at San Pio Hospital of Vasto (Italy). During follow-up, clinical evaluations were performed as part of routine care. Written informed consent was obtained from all participants in accordance with the Declaration of Helsinki.

### Clinical assessments

- *Motor Symptoms*: Motor severity was assessed using the MDS-Unified Parkinson’s Disease Rating Scale Part III (MDS-UPDRS III).
- *Cognitive Function*: Cognitive function was measured using the Montreal Cognitive Assessment (MoCA). A cut-off of 19.015, validated for the Italian population, was used to define definite cognitive impairment (17). Evaluations were performed at baseline and at 5-year follow-up
- *Psychiatric Symptoms*: The Neuropsychiatric Inventory (NPI) was employed to evaluate psychiatric symptoms. The One-Question mayo Sleep Questionnaire item was used to assess the presence of nighttime sleep disturbances.
- *Premotor Symptoms*: Retrospective structured interviews assessed the presence of mood disorders (depression and anxiety) and RBD predating motor symptoms by at least 3 years. Whenever available, information provided by patients was corroborated through caregiver interviews and review of previous medical records to improve the reliability of retrospective symptom ascertainment.
- *REM Sleep Behavior Disorder* (*RBD*): RBD was assessed using the One-Question Mayo Sleep Questionnaire, a validated screening tool with high sensitivity for identifying probable RBD. The instrument provides a dichotomous classification (presence/absence of dream-enactment behaviors) and does not generate a severity score. In this study, it was used as a screening measure to identify premotor sleep disturbances.

### Imaging and group classification

All patients underwent DAT-SPECT imaging using [123I]FP-CIT according to EANM guidelines [16]. Semi-quantitative analysis was performed by manually placing regions of interest (ROIs) over the bilateral putamen and caudate nuclei, with the occipital cortex used as a reference region. Striatal binding ratios (SBRs) were calculated accordingly.

Reduced dopaminergic uptake was defined based on visual assessment supported by semi-quantitative SBR values below the age-adjusted reference range, in line with established clinical practice (16).

Patients were classified into two groups based on the topographical pattern of dopaminergic denervation:

- P group (putaminal pattern): reduced uptake in the putamen with relatively preserved uptake in the caudate nuclei
- PC group (putaminal + caudate pattern): reduced uptake involving both putamen and caudate nuclei

Group classification was based on consensus visual interpretation supported by semi-quantitative analysis.

### Statistical analysis

Continuous variables were analyzed using Student’s t-test or Mann–Whitney U test, and categorical variables with chi-square or Fisher’s exact test, as appropriate. Data distribution was evaluated using the Shapiro–Wilk test. Statistical significance was defined as *p* < 0.05. Analyses were performed using SPSS version 14.0 (IBM Corp.).

Changes in LEDD over time were analyzed using a repeated-measures approach with time (baseline vs. follow-up) as the within-subject factor and group (P group vs. PC group) as the between-subject factor.

## Results

Demographic and clinical characteristics are shown in Table 1.

**Table 1:** Demographic and clinical characteristics at baseline.

Variable	P group (*n* = 12)	PC group (*n* = 16)	*p*-value
Age (years)	64.31 ± 8.5	62.33 ± 8.65	n.s.
Gender (M/F)	5/7	10/6	n.s.
Disease duration (years)	3.0 ± 2.68	1.76 ± 1.09	n.s.
UPDRS III score	20.44 ± 5.96	31.29 ± 8.16	<0.0001
Rigid phenotype (%)	12.5	66.7	<0.0001
Tremor-dominant (%)	56.3	4.8	<0.0001
Mixed phenotype (%)	31.3	28.6	n.s.
Premotor psychiatric symptoms (%)	12.5	47.5	0.001
Psychiatric symptoms at baseline (%)	31.3	71.4	<0.01

Twelve patients were classified as P group (5 males). Sixteen patients were classified as PC group (10 males) with involvement of the left caudate in 11 patients. There were no significant group differences in terms of age, gender, or disease duration, although PC patients exhibited significantly higher UPDRS III scores at baseline (*t*(25) = 4.06, *p* < 0.001, 95% CI [5.35, 16.35], Hedges’ *g* = 1.44), indicating more severe motor impairment in this group.

Patients were classified into the tremor-dominant (TD), mixed or akinetic-rigid (AR) subtypes, according to previous reports (18, 19). The AR phenotype was predominant in the PC group (66.7%) compared to the P group (12.5%; Fisher’s exact test, *p* < 0.001, odds ratio = 24.2, 95% CI [2.4–243.6]). Conversely, the TD phenotype was more frequent in the P group (56.3%) than in PC group (4.8%; Fisher’s exact test, *p* < 0.001, odds ratio = 21.0, 95% CI [2.0–217.4]). The proportion of mixed phenotype did not differ significantly between groups (31.3% vs. 28.6%; Fisher’s exact test, *p* = 1.00).

RBD was rarely observed in both groups, occurring in 2 patients in the P group and 4 patients in the PC group, with no significant difference between groups.

Premotor psychiatric symptoms, such as anxiety and depression, present at least 3 years before the motor symptoms were more prevalent in PC group (47.5%) than in P group (12.5%; Fisher’s exact test, *p* = 0.03, odds ratio = 11.0, 95% CI [1.1–112.0]).

At baseline, psychiatric symptoms (generalized anxiety, panic attacks, phobia, OCD, moderate to severe depression) were more frequent in PC patients (71.4%) than in P group (31.3%; Fisher’s exact test, *p* = 0.03, odds ratio = 6.9, 95% CI [1.3–36.3]).

At baseline, 5 of 16 PC patients (32%) exhibited MoCA scores below the cognitive impairment cutoff (<19.015), compared to none of the 12 P patients (0%; Fisher’s exact test, *p* = 0.045). At 5-year follow-up, 13 of 16 PC patients (83%) scored below the cutoff, compared to only 1 of 12 PD-p patients (9%; Fisher’s exact test, *p* < 0.001, odds ratio = 30, 95% CI [3.3–272]).

Both P and PC groups showed progressive worsening of UPDRS III scores over 5 years (mean ΔUPDRS ± SD: PD-*p* = 8.32 ± 7.32; PD-pc = 11.89 ± 6.42). Although the increase was greater in the PC group, the difference was not statistically significant (*t*(26) = 0.66, *p* = 0.5093, Cohen’s *d* = −0.25), suggesting a small effect size. Both groups displayed a generally linear progression over time.

Hallucinations were found in four PC patients at 5-year follow up: they were described as simple and mainly zoomorphic and with retained insight, occurring prevalently at home. No hallucinations were observed in P group at 5-year follow up.

LEDD increased over time in both groups. In the P group mean LEDD rose from 500 mg at baseline to 600 mg at follow-up, while in the PC group it increased from 680 to 850 mg. Across both time points, PC patients showed consistently higher LEDD values compared with P patients.

Results at follow up are shown in Table 2.

**Table 2:** Cognitive outcomes (MoCA) at baseline and 5-year follow-up.

Time point	P group (*n* = 12)	PC group (*n* = 16)	*p* value
Baseline	27.25 ± 2.03	22 ± 4.3	<0.01
5-year follow-up	23.58 ± 2.39	17.20 ± 2.83	<0.001

## Discussion

This longitudinal study demonstrates that early dopaminergic denervation involving the caudate nucleus identifies a subgroup of patients with parkinsonism characterized by a more severe clinical phenotype, including greater motor impairment, higher neuropsychiatric burden, and a markedly accelerated cognitive decline over 5 years. These findings are consistent with previous PET and SPECT studies showing that caudate dopaminergic dysfunction is associated with cognitive impairment and accelerated progression in early PD (9, 10, 12, 20). Together, this evidence support the hypothesis that the topographical distribution of striatal dopaminergic degeneration contributes to clinical heterogeneity within Lewy body disorders and may have important prognostic implications (6, 9, 20).

Importantly, the clinical profile observed in patients with caudate involvement, including early cognitive impairment, rapid progression, and the emergence of visual hallucinations, suggests that this subgroup may lie toward the cognitive and neuropsychiatric end of the Lewy body disease spectrum. This interpretation aligns with imaging evidence indicating that early caudate involvement reflects more diffuse Lewy body pathology and predicts transition toward cognitive phenotypes (12, 13). While all patients fulfilled clinical diagnostic criteria for Parkinson’s disease at baseline, the longitudinal evolution of this subgroup raises the possibility that some individuals may have progressed toward a dementia with Lewy bodies (DLB)-like phenotype or represent cases of overlapping Lewy body pathology. This impression was driven by the combination of progressive cognitive deterioration and the development of recurrent visual hallucinations. However, because a formal longitudinal reassessment according to established DLB diagnostic criteria was not prospectively performed, no definitive diagnostic reclassification could be made. This interpretation is consistent with the recognized diagnostic overlap between Parkinson’s disease, Parkinson’s disease dementia, and DLB, particularly in early disease stages (1).

At baseline, patients with caudate involvement already exhibited lower cognitive performance, with a substantial proportion showing impaired MoCA scores despite relatively short disease duration. This pattern is atypical for early idiopathic Parkinson’s disease, in which cognition is usually relatively preserved. Over follow-up, these patients showed a steeper cognitive decline compared to those with isolated putaminal involvement, suggesting a more aggressive neurodegenerative trajectory (2, 18).

The occurrence of visual hallucinations in a subset of patients with caudate involvement further strengthens this interpretation. Although hallucinations may occur in advanced Parkinson’s disease or as a treatment-related complication, they are also a core clinical feature of DLB and are strongly associated with more diffuse cortical and limbic involvement (1). In the context of early cognitive impairment and caudate dopaminergic dysfunction, their presence supports positioning these patients within a broader Lewy body disease spectrum rather than within a purely motor-defined Parkinsonian subtype.

From a neurobiological perspective, these findings are consistent with the functional role of the caudate nucleus within associative and limbic cortico-striatal circuits. Dopaminergic dysfunction in this region has been associated with executive dysfunction, behavioral changes, and cognitive decline in Parkinsonian disorders (9, 10, 20). Early caudate involvement may therefore reflect more widespread synuclein pathology affecting both motor and non-motor networks.

Our results also contribute to the ongoing discussion on motor subtypes in Parkinson’s disease. Although the akinetic-rigid phenotype was more frequent in patients with caudate involvement, motor subtype classifications are known to be unstable over time and of limited prognostic value when considered in isolation (18, 19, 21). In contrast, imaging-based stratification using DAT-SPECT may provide a more biologically grounded approach to capturing disease heterogeneity.

A key implication of this study is that DAT-SPECT-derived striatal patterns may have prognostic relevance beyond their diagnostic utility. However, DAT-SPECT cannot differentiate Parkinson’s disease from other Lewy body disorders, including DLB (1, 7). Therefore, imaging findings should be interpreted as markers of dopaminergic system involvement rather than disease-specific signatures, and must be integrated with longitudinal clinical assessment.

Several limitations should be acknowledged. The relatively small sample size limits generalizability and precludes adjustment for potential confounders such as age, comorbidities, and baseline disease severity. Consequently, part of the observed differences in motor progression may reflect the greater baseline motor impairment of the PC group rather than the topographical pattern of dopaminergic denervation alone. Similarly, although the marked cognitive divergence between groups strongly suggests a distinct clinical trajectory, the contribution of unmeasured confounding factors cannot be completely excluded. Premotor psychiatric symptoms were assessed retrospectively and are therefore subject to recall bias. In addition, formal longitudinal diagnostic re-evaluation was not systematically performed, and some patients may have met criteria for DLB or mixed Lewy body pathology during follow-up. This diagnostic uncertainty, however, reinforces a spectrum-based interpretation of the findings rather than discrete categorical separation.

A further limitation of the present study is the absence of an extensive neuropsychological battery. However, the MoCA is widely used in longitudinal PD research and is sensitive to fronto-subcortical decline, which represents the most common cognitive trajectory in PD. Our aim was to evaluate cognitive trajectories rather than detailed cognitive profiles. The consistent 5-year decline observed in the PC group suggests that the association between caudate denervation and cognitive worsening is robust even when measured through a global cognitive screening tool.

In conclusion, early caudate dopaminergic denervation identifies a parkinsonian phenotype associated with rapid cognitive decline and prominent neuropsychiatric features. Rather than representing a distinct subtype of idiopathic Parkinson’s disease, this pattern may reflect a position along the continuum of Lewy body disorders, spanning Parkinson’s disease and dementia with Lewy bodies. These findings highlight the potential role of DAT-SPECT in early risk stratification and underscore the importance of longitudinal, biomarker-driven approaches to understanding disease heterogeneity in parkinsonism.

## Data Availability

The raw data supporting the conclusions of this article will be made available by the authors, without undue reservation.

## References

[1] McKeith IG Boeve BF Dickson DW Halliday G Taylor JP Weintraub D. Diagnosis and management of dementia with Lewy bodies: fourth consensus report of the DLB consortium. Neurology. (2017) 89:88–100. 10.1212/WNL.0000000000004058 PMC5496518 28592453

[2] Aarsland D Brønnick K Ehrt U De Deyn PP Tekin S Emre M. Neuropsychiatric symptoms in patients with Parkinson’s disease and dementia: frequency, profile and associated caregiver stress. J Neurol Neurosurg Psychiatry. (2007) 78:36–42. doi: 10.1136/jnnp.2005.083113, 16820421 PMC2117797

[3] Chaudhuri KR Healy DG Schapira AH. Non-motor symptoms of Parkinson’s disease: diagnosis and management. Lancet Neurol. (2006) 5:235–45. doi: 10.1016/S1474-4422(06)70373-8, 16488379

[4] Schapira AHV Chaudhuri KR Jenner P. Non-motor features of Parkinson disease. Nat Rev Neurosci. (2017) 18:435–50. doi: 10.1038/nrn.2017.62, 28592904

[5] Martinez-Martin P Rodriguez-Blazquez C Kurtis MM Chaudhuri KR. The impact of non-motor symptoms on health-related quality of life of patients with Parkinson’s disease. Mov Disord. (2011) 26:399–406. doi: 10.1002/mds.23462, 21264941

[6] Weintraub D Moberg PJ Duda JE Katz IR Stern MB. Recognition and treatment of depression in Parkinson's disease. J Geriatr Psychiatry Neurol. (2003) 16:178–83. doi: 10.1177/0891988703256053, 12967062

[7] Bohnen NI Albin RL. The cholinergic system and Parkinson disease. Behav Brain Res. (2011) 221:564–73. doi: 10.1016/j.bbr.2009.12.048, 20060022 PMC2888997

[8] Alexander GE DeLong MR Strick PL. Parallel organization of functionally segregated circuits linking basal ganglia and cortex. Annu Rev Neurosci. (1986) 9:357–81. doi: 10.1146/annurev.ne.09.030186.002041, 3085570

[9] Jokinen P Brück A Aalto S Forsback S Parkkola R Rinne JO. Impaired cognitive performance in Parkinson's disease is related to caudate dopaminergic hypofunction: a PET study. NeuroImage. (2009) 45:386–92. 10.1016/j.parkreldis.2008.03.005 18434233

[10] Nobili F Campus C Arnaldi D De Carli F Cabassi G Brugnolo A. Cognitive-nigrostriatal relationships in de novo, drug-naïve Parkinson’s disease patients: a [123I]FP-CIT SPECT study. Mov Disord. (2010) 25:35–43. 10.1002/mds.22899 20058228

[11] Christopher L Marras C Duff-Canning S Yuko K Robert C Isabelle B . Combined striatal and cortical dopamine dysfunction predicts cognitive decline in Parkinson’s disease. Brain. (2014) 137:1700–15.

[12] Nobili F Campus C Arnaldi D De Carli F Cabassi G Brugnolo A . Longitudinal caudate dopaminergic dysfunction predicts cognitive decline in Parkinson’s disease. Neurobiol Aging. (2015) 36:821–7.

[13] Brück A Kurki T Kaasinen V Vahlberg T Rinne JO. Caudate nucleus atrophy and cognitive decline in Parkinson’s disease. J Neurol Neurosurg Psychiatry. (2015) 86:608–14. 10.1136/jnnp.2003.031237 PMC1738757 15377698

[14] Melzer TR Watts R MacAskill MR Pitcher TL Livingston L Keenan RJ . Grey matter atrophy in cognitively impaired Parkinson's disease. J Neurol Neurosurg Psychiatry. (2012) 83:188–94. doi: 10.1136/jnnp-2011-300828, 21890574

[15] Brück A Kurki T Kaasinen V Vahlberg T Rinne JO. Hippocampal and prefrontal atrophy in patients with early non-demented Parkinson’s disease is related to cognitive impairment. J Neurol Neurosurg Psychiatry. (2004) 75:1467–9. doi: 10.1136/jnnp.2003.031237, 15377698 PMC1738757

[16] Booij J Knol RJA. SPECT imaging of the dopamine transporter: an overview. Handb Clin Neurol. (2021) 183:231–48.

[17] Choi SM Kim BC Cho BH Kang KW Choi KH Kim JT . Validity and diagnostic accuracy of the Italian version of the Montreal cognitive assessment (MoCA) in non-demented Parkinson’s disease patients. Neurol Sci. (2023) 44:5793–802.

[18] Kang GA Bronstein JM Masterman DL Redelings M Crum JA Ritz B. Clinical characteristics in early Parkinson's disease in a Central California population-based study. Mov Disord. (2005) 20:1133–42. doi: 10.1002/mds.20513, 15954133 PMC3643967

[19] Choi SM Kim BC Cho BH Kang KW Choi KH Kim JT . Comparison of two motor subtype classifications in de novo Parkinson's disease. Parkinsonism Relat Disord. (2018) 54:74–8. doi: 10.1016/j.parkreldis.2018.04.021, 29703644

[20] Rinne JO Portin R Ruottinen H Nurmi H Bergman J Haaparanta M. Cognitive impairment and the brain dopaminergic system in Parkinson disease: [18F]fluorodopa PET study. Neurology. (2000) 54:401–6. 10.1001/archneur.57.4.470 10768619

[21] Eggers C Pedrosa DJ Kahraman D Maier F Fink GR Timmermann L. Different progression in Parkinson subtypes: an analysis of 17 years of longitudinal data. Mov Disord. (2012) 27:1041–7.

